# Severe Presentation of Cannabinoid Hyperemesis Syndrome With Mixed Acid–Base Disorder and Cardiac Complications: A Case Report

**DOI:** 10.1155/carm/8161294

**Published:** 2025-05-21

**Authors:** Giovanni Mantelli, Armando Fiore, Caterina Barberi, Barbara Zaia, Giorgio Ricci, Massimo Carollo, Fabio Malalan

**Affiliations:** ^1^Emergency Department, APSS-Provincia Autonoma di Trento, Rovereto, Italy; ^2^National Poison Center, Azienda Ospedaliera Universitaria Integrata, Verona, Italy; ^3^Department of Primary Care, ULSS 1 Dolomiti, Belluno, Italy; ^4^Department of Diagnostics and Public Health, University of Verona, Verona, Italy

**Keywords:** cannabinoid hyperemesis syndrome, cannabinoids, emergency medicine, illicit drugs, poison control centers, toxicology

## Abstract

**Background:** Cannabinoid hyperemesis syndrome (CHS) is a condition characterized by cyclic abdominal pain, vomiting, and nausea, primarily affecting adolescents and adults with a history of chronic cannabis use. The diagnosis of CHS is clinical, with symptom resolution upon cannabis cessation considered pathognomonic. The overlap of CHS symptoms with other conditions complicates the differential diagnosis, particularly in emergency settings.

**Case Presentation:** We report an unusual case of a 28-year-old man admitted to the Emergency Department of Rovereto (Italy) with limb paresthesia and agitation. Initial evaluation revealed indirect clinical signs of hypocalcemia, and QTc prolongation and severe hypokalemia on electrocardiogram. The arterial blood gas analysis suggested mixed acid–base disturbances. His symptoms improved with aggressive electrolyte correction, benzodiazepine administration, magnesium sulfate administration, and fluid resuscitation. Given the significant risk of arrhythmias, antiemetics known to prolong QTc, such as dopamine antagonists, were contraindicated, and midazolam was used as an alternative for symptom control (both nausea and agitation).

**Conclusion:** This case underscores the importance of recognizing CHS as a potential etiology in patients with recurrent vomiting and a history of chronic cannabis use, even in the presence of atypical findings such as profound electrolyte imbalances and cardiac abnormalities. Given the protracted recovery period associated with CHS, early identification and patient education regarding cannabis cessation are crucial in preventing recurrent episodes.

## 1. Background

Cannabinoid hyperemesis syndrome (CHS) is a condition characterized by cyclic episodes of intense abdominal pain, severe nausea, and recurrent vomiting in individuals with a history of chronic and regular cannabis use [1, 2]. Symptoms typically arise within 24 h of the last cannabis use, distinguishing CHS from cannabis withdrawal, where symptoms usually manifest after 24–72 h [3]. Abdominal pain associated with CHS is often intense, diffuse, and predominantly localized to the periumbilical or epigastric regions, frequently accompanied by pallor and diaphoresis [3, 4]. These features, alongside the severity of vomiting—up to 30 episodes per day—help differentiate CHS from other conditions in its differential diagnosis [3, 4]. While the majority of patients maintain normal bowel habits, a minority report diarrhea and chills during episodes [3, 4]. Interestingly, relief from symptoms is often achieved through hot showers or baths, a behavior so pronounced in some cases that it leads to compulsive bathing, with patients taking several showers daily [3–5].

The resolution of symptoms, which may persist for 6–12 months, upon cannabis cessation is a key diagnostic criterion for CHS, although symptomatic relief may take several days. Patients are generally asymptomatic between episodes, which can span weeks to months, adding complexity to the recognition and diagnosis of the syndrome [3, 4]. The prevalence of CHS is challenging to estimate precisely, but it is increasingly recognized as a significant public health concern [6]. Indeed, CHS is an underdiagnosed condition, and observational studies suggest that it may affect up to 50% of individuals presenting to emergency departments with recurrent vomiting and a history of cannabis use [7, 8].

The underdiagnosis of CHS can be attributed to several factors. First, there is often a lack of awareness about the condition among healthcare providers, particularly in regions where cannabis use is less prevalent or less openly discussed [9–11]. Second, the clinical presentation of CHS overlaps with that of other conditions, such as cyclic vomiting syndrome, gastrointestinal disorders, or even substance withdrawal syndromes, complicating the differential diagnosis [3, 9–11]. Third, patients themselves may underreport cannabis use due to stigma or fear of legal repercussions, leading to diagnostic delays [9–11]. Finally, in emergency settings, the focus on stabilizing acute symptoms may deprioritize the exploration of underlying causes such as CHS [3, 4].

Here, we present the case of a young man who arrived at the Emergency Department of Rovereto (Italy) with an atypical clinical presentation, highlighting the challenges of diagnosing CHS in the presence of unusual symptoms and severe metabolic derangements.

## 2. Case Report

A 28-year-old Caucasian man presented to the Emergency Department in a state of agitation and distress, reporting tingling sensations diffusely distributed throughout his body, more pronounced in the extremities of all four limbs and the perioral region. During the measurement of blood pressure, a positive Trousseau's sign and mild Chvostek's sign were observed bilaterally. The patient reported experiencing similar symptoms multiple times in the past months, but he described the current episode as significantly more severe and unresponsive to his usual coping mechanism of taking hot showers. The patient denied any significant past medical conditions and reported an allergy to ketoprofen.

**Figure 1:**
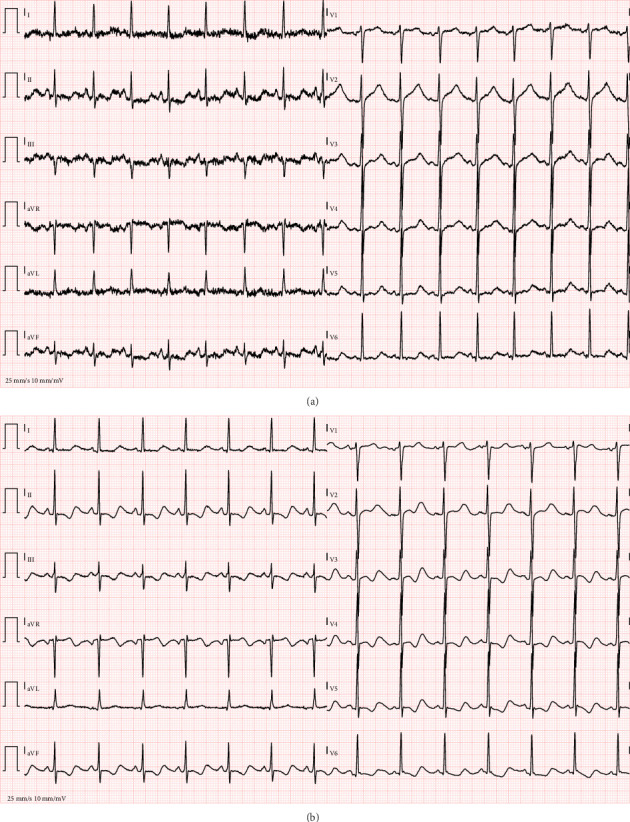
(a) First electrocardiogram: prolonged QT interval, presence of U waves, and ST-T segment irregularities, consistent with severe hypokalemia and mild hypocalcemia. (b) Second electrocardiogram, 45 min later.

At triage, the patient was found to be hypertensive, tachycardic, and mildly febrile (37.8°C). A physical examination revealed epigastric tenderness, which the patient associated with several days of recurrent vomiting. The first electrocardiogram (ECG) showed sinus tachycardia with severe QT prolongation (480 ms, QTc: Bazett 601 ms, Hodges 540 ms) (Figure 1(a)), raising concerns about potential arrhythmias. A second ECG, 45 min later, showed worsening of the QT prolongation (518 ms, QTc: Bazett 609 ms, Hodges 558 ms) (Figure 1(b)). The arterial blood gas analysis indicated a mixed acid–base disorder, characterized by marked metabolic alkalosis with a contribution from respiratory alkalosis, as suggested by the lower-than-expected pCO_2_ level (Table 1). In addition, the patient's elevated anion gap (20.5 mmol/L) and initial lactic acid level (7.0 mmol/L) suggest the presence of a metabolic acidosis component, likely due to lactic acidosis secondary to severe dehydration and prolonged vomiting. However, the arterial blood gas analysis was dominated by metabolic alkalosis (pH 7.61), with the concurrent metabolic acidosis likely masked by the bicarbonate elevation. Moreover, arterial blood gas analysis showed marked hypochloremia, severe hypokalemia, and mild hypocalcemia (Table 1). The patient was promptly placed on a multiparameter monitor, and Automated External Defibrillator (AED) adhesive pads were applied due to the increased risk of malignant arrhythmias.

A Point-of-Care Ultrasound (POCUS) was performed, revealing signs of severe dehydration, including a completely collapsed inferior vena cava and a “kissing walls” pattern in the left ventricle, indicative of significant volume depletion. Initial resuscitative measures included a 250 mL bolus of intravenous (i.v.) normal saline, followed by a continuous infusion of 2 L over 2 hours. Magnesium sulfate (4 g i.v.) was administered due to the proarrhythmic risk associated with hypokalemia and QTc prolongation. Potassium replacement therapy was initiated promptly, as the corrected potassium levels remained critically low, even after adjusting for pH-related shifts.

In a few hours, the patient's nausea progressively worsened, exacerbating his agitation. Considering the risk of *torsades de pointes* associated with QTc prolongation, antiemetics such as haloperidol, droperidol, or metoclopramide were contraindicated. Instead, repeated boluses of midazolam (5 mg i.v.) were administered, effectively controlling both the patient's agitation and nausea. During this period, the patient disclosed a history of regular cannabis use and recurrent episodes of cyclic vomiting. This information, combined with his clinical findings, supported the diagnosis of CHS.

**Figure 2:**
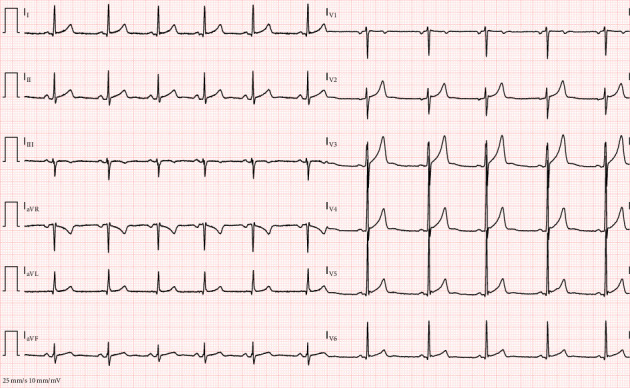
Second electrocardiogram after electrolyte imbalance correction: normalization of the QT interval and resolution of ST-T segment irregularities.

Over his eight-hour stay in the Emergency Department intensive observation unit, the patient received aggressive electrolyte replacement, including 80 mEq of i.v. potassium chloride and an additional 16 mEq administered orally. Calcium gluconate was administered to address mild hypocalcemia, and spontaneous oral fluid intake resumed shortly after the administration of midazolam. By the end of his stay, with correction of the electrolyte imbalances and administration of a total of 4 L of fluids via i.v. and oral routes, the patient showed significant clinical improvement. A repeat ECG at discharge demonstrated a normal sinus rhythm with restoration of QT (382 ms, QTc: Bazett 398 ms, Hodges 391 ms) (Figure 2). Despite residual hypokalemia, the patient chose to self-discharge against medical advice. He was counseled on the importance of cannabis cessation and referred for follow-up care to address the underlying cause of his symptoms.

## 3. Discussion

The pathogenesis of CHS remains incompletely understood, likely involving a multifactorial interplay of alterations in cannabinoid metabolism, cumulative dose exposure, and receptor tolerance leading to dysregulation of the endocannabinoid system. Cannabinoid receptor type 1 (CB1) plays a key role in gastric secretion, motility, sensation, and inflammation. CB1 receptors also inhibit the hypothalamic–pituitary–adrenal axis and the sympathetic nervous system's stress response [12]. Paradoxically, while cannabinoids are often utilized for their antiemetic properties, chronic cannabis use may result in CB1 receptor desensitization and downregulation, potentially explaining the persistence of symptoms in CHS patients even during the recovery phase [13].

Tetrahydrocannabinol (THC), the principal psychoactive compound in cannabis, functions not only as an agonist of CB1 and CB2 receptors but also as an agonist of the transient receptor potential vanilloid 1 (TRPV1) channel, also known as the capsaicin receptor. Prolonged cannabis use may overstimulate TRPV1, contributing to the cyclic nausea, vomiting, and abdominal discomfort characteristic of CHS [14]. Capsaicin, a potent TRPV1 agonist, has been investigated as a symptomatic treatment for CHS [15, 16]. In addition, THC-induced splanchnic vasodilation coupled with cutaneous vasoconstriction (a phenomenon referred to as “cutaneous steal syndrome”) may explain the symptomatic relief reported by many CHS patients during hot showers, likely mediated via peripheral TRPV1 activation [14, 16].

The diagnosis of CHS is clinical, based primarily on a patient's history of chronic cannabis use and recurrent episodes of severe abdominal pain and vomiting [17]. Even if typical symptoms are present, it is essential to exclude other serious conditions through a thorough medical history, physical examination, and appropriate diagnostic testing, such as laboratory evaluations and abdominal imaging [17]. Ultrasound can serve as a first-line adjunct to rule out differential diagnoses such as biliary colic, pancreatitis, or intestinal obstruction [3, 4].

Acute treatment of CHS involves aggressive fluid resuscitation and antiemetic therapy, and dopaminergic antagonists such as metoclopramide or haloperidol are first-line antiemetic agents [3, 4, 18]. Benzodiazepines, such as lorazepam, may serve as adjunctive therapy for patients presenting with agitation [3, 4]. In our case, given the prolonged QTc interval, the use of first-line antiemetics was contraindicated due to the risk of *torsades de pointes*. As such, midazolam was administered for its dual anxiolytic and antiemetic properties. Repeated i.v. boluses of 5 mg midazolam effectively alleviated the patient's nausea and agitation, providing symptom control without exacerbating the cardiac risks.

In our case, fluid resuscitation played a pivotal role in breaking the vicious cycle of vomiting and electrolyte disturbances. One of the primary focuses was correcting hypokalemia, which was assumed to be secondary to recurrent vomiting and potassium shifts from the intracellular to the extracellular compartment. The calculated 48 mmol of potassium deficit, taking into account the patient's target level of 4.5 mmol/L and the body weight of the patient (70 kg, with an estimated total body water of 45 L), was considered to be a small amount. As such, an i.v. potassium supplementation of 80 mmol was followed by oral potassium supplementation to address any residual needs. The decision to limit i.v. administration was guided by patient preferences and the practicality of further correction through oral intake, which would avoid the need for prolonged hospitalization or central venous access. Regarding calcium levels, although laboratory testing revealed only mild hypocalcemia, clinical signs such as positive Trousseau's and Chvostek's signs were nonetheless present, and this prompted calcium supplementation. These findings may also be influenced by the potential of cannabinoids to modulate neuromuscular excitability and calcium signaling, possibly amplifying clinical manifestations even in cases of borderline hypocalcemia [19–21]. Lastly, the decision to administer magnesium sulfate was guided by its potential role in stabilizing cardiac membranes and preventing arrhythmias, particularly in the context of severe hypokalemia and QTc prolongation [22–25].

After achieving clinical stabilization, the patient elected to leave the hospital against medical advice. This patient's decision underscores the ongoing challenges in managing CHS, particularly when long-term cannabis cessation is essential for recovery [3, 4]. However, it represented a missed opportunity not only to address the risk of symptom recurrence but also to initiate therapeutic education and structured intervention. Although healthcare providers are not responsible for a patient's autonomous decision to self-discharge, such actions can hinder the implementation of comprehensive care plans, including addiction counseling and medical, psychological, and social follow-up support [9, 26, 27]. This scenario highlights the importance of early and empathetic communication about the risks of continued cannabis use and the benefits of cessation, as well as the need for accessible outpatient resources (such as, in Italy, addiction services called *Servizi per le Dipendenze patologiche*, SerD) to support patients after discharge [9, 26, 27].

## 4. Conclusions

This case highlights a severe presentation of CHS complicated by mixed acid–base disorder, severe hypokalemia, and QTc prolongation. The management of this case required a multidisciplinary approach, emphasizing the critical importance of correcting both the underlying cause and the resultant complications. In addition, it highlights the utility of benzodiazepines as a safe and effective antiemetic alternative in patients where standard antiemetics pose significant risks. Great awareness of CHS among general practitioners and emergency clinicians is essential to avoid diagnostic delays, facilitate early intervention, and improve patient outcomes. Equally important is the implementation of structured outpatient follow-up and addiction support services, which are crucial to promoting long-term cannabis cessation and preventing recurrence.

## Figures and Tables

**Table 1:** Laboratory tests at emergency department admission and during hospital stay.

	At ED admission (venous)	Four hours (arterial)	Six hours (venous)	Day 6 (venous)
pH	7.61	7.52	7.52	7.368
pCO_2_ (mmHg)	32.0	38.8	36.6	44.9
pO_2_ (mmHg)	25.6	94.1	49.1	32.9
Potassium (mmol/L)	2.9	2.7	3.0	4.0
Calcium (ionized) (mg/dL)	4.14	4.06	4.20	4.94
Sodium (mmol/L)	132	130	132	138
Chlorine (mmol/L)	79	88	92	105
Lactates (mmol/L)	7.0	0.9	1.1	0.9
Anion gap (mmol/L)	20.5	10.4	10.5	8.1
Bicarbonates (mmol/L)	32.4	31.7	29.8	25.2

Abbreviation: ED, Emergency Department.

## Data Availability

The data that support the findings of this study are available on request from the corresponding author. The data are not publicly available due to privacy or ethical restrictions.
